# Syntheses, crystal structures and Hirshfeld surface analyses of (*E*)-1-[2,2-di­chloro-1-(2,3-di­meth­oxyphen­yl)ethen-1-yl]-2-phenyl­diazene and (*E*)-1-(4-chloro­phen­yl)-2-[2,2-di­chloro-1-(2,3-di­meth­oxy­phen­yl)ethen-1-yl]diazene

**DOI:** 10.1107/S2056989026000137

**Published:** 2026-01-13

**Authors:** Namiq Q. Shikhaliyev, Naila Mammadova, Gulnar T. Atakishiyeva, Peri A. Huseynova, Gulnara V. Babayeva, Gulnaz A. Mirzayeva, Mehmet Akkurt, Ajaya Bhattarai

**Affiliations:** a Department of Chemical Engineering, Baku Engineering University, Khirdalan City, 120 AZ0101 Hasan Aliyev Street, Baku, Azerbaijan; b Organic Chemistry Department, Baku State University, Z. Khalilov str. 23, AZ 1148 Baku, Azerbaijan; c Chemistry Department, Nakhchivan State University, University Campus, AZ7012, Nakhcivan, Azerbaijan; d Department of Analytical and Organic Chemistry, Azerbaijan State Pedagogical University, 68 Uzeyir Hajibeyli str., Baku AZ1000, Azerbaijan; e Department of Chemical Technology, Recycling and Ecology, Azerbaijan Technical University, Baku, Azerbaijan, H. Javid ave 25, AZ 1073 Baku, Azerbaijan; f Department of Physics, Faculty of Sciences, Erciyes University, 38039 Kayseri, Türkiye; g Department of Chemistry, M.M.A.M.C (Tribhuvan University) Biratnagar, Nepal; Institute of Chemistry, Chinese Academy of Sciences

**Keywords:** crystal structure, C—H⋯π inter­actions, C—Cl⋯π inter­actions, van der Waals inter­actions, Hirshfeld surface analysis

## Abstract

The crystal structures and Hirshfeld surface analyses of two similar azo compounds are reported. (*E*)-1-[2,2-di­chloro-1-(2,3-di­meth­oxy­phen­yl)ethen-1-yl]-2-phenyl­diazene, C_16_H_14_Cl_2_N_2_O_2_, (**I**), crystallizes in space group *P*2_1_/*c* with *Z* = 4, and (*E*)-1-(4-chloro­phen­yl)-2-[2,2-di­chloro-1-(2,3-di­meth­oxy­phen­yl)ethen-1-yl]diazene, C_16_H_13_Cl_3_N_2_O_2_, (**II**), in the space group *P*1 with *Z* = 4. In the crystal structure of (**I**), the mol­ecules form layers parallel to the (010) plane through C—H⋯π and C—Cl⋯π inter­actions and van der Waals inter­actions between these layers consolidate the packing. There are two symmetry-independent mol­ecules in the asymmetric unit of (**II**). In the crystal, mol­ecules are connected by C—H⋯O and C—H⋯Cl hydrogen bonds, forming a three-dimensional network. C—Cl⋯π inter­actions also contribute to the packing. The inter­molecular contacts in the crystals (**I**) and (**II**) were analysed using Hirshfeld surface analysis and two-dimensional fingerprint plots.

## Abstract 2

The crystal structures and Hirshfeld surface analyses of two similar azo compounds are reported. In the first, the mol­ecules form layers parallel to the (010) plane through C—H⋯π and C—Cl⋯π inter­actions and van der Waals inter­actions between these layers consolidate the packing. In the other, the mol­ecules are connected by C—H⋯O and C—H⋯Cl hydrogen bonds, forming a three-dimensional network. C—Cl⋯π inter­actions also contribute to the packing.

## 1. Chemical context

**Figure 1:**
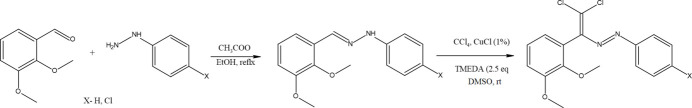
Reaction scheme for compounds (**I**) and (**II**).

Azo dyes continue to attract considerable attention due to their wide applications in the textile (O’Neill *et al.*, 2000 ▸; Garg *et al.*, 2017 ▸), optical (Al-Mudhaffer *et al.*, 2016 ▸; Mohr & Wolfbeis, 1994 ▸), and biological fields (Khan *et al.*, 2021 ▸; Singh & Singh, 2017 ▸). The presence of functional groups in the obtained compounds provides broad opportunities for further chemical transformations and structural modifications. In this paper, we report the synthesis of two new di­chlorodi­aza­dienes, namely (*E*)-1-[2,2-di­chloro-1-(2,3-di­meth­oxy­phen­yl)ethen-1-yl]-2-phenyl­diazene, C_16_H_14_Cl_2_N_2_O_2_, (**I**), and (*E*)-1-(4-chloro­phenyl)-2-[2,2-di­chloro-1-(2,3-di­meth­oxy­phen­yl)ethen-1-yl]dia­zene, C_16_H_13_Cl_3_N_2_O_2_, (**II**). These compounds were synthesized in two steps starting from 2,3-di­meth­oxy­benzaldehyde and phenyl­hydrazine and its chloro-substituted derivative. In the first step, the corresponding Schiff bases were obtained by condensation in ethanol under reflux in the presence of acetic acid. In the second step, the resulting hydrazones were converted into the target azo dyes by reaction with carbon tetra­chloride in DMSO at room temperature in the presence of a CuCl_2_ catalyst and tetra­methyl­ethylenedi­amine (TMEDA) (Fig. 1 ▸).

The formation of a di­chloro­ethenyl fragment and an azo (–N=N–) chromophore within the same mol­ecular system significantly enhances the functional diversity of the synthesized compounds. Such structural features not only affect their electronic and optical properties, but also enable their participation in various inter­molecular inter­actions in the solid state. Therefore, in addition to the synthesis, detailed single-crystal X-ray diffraction and Hirshfeld surface analyses were performed to investigate the mol­ecular and supra­molecular structures of compounds (**I**) and (**II**).
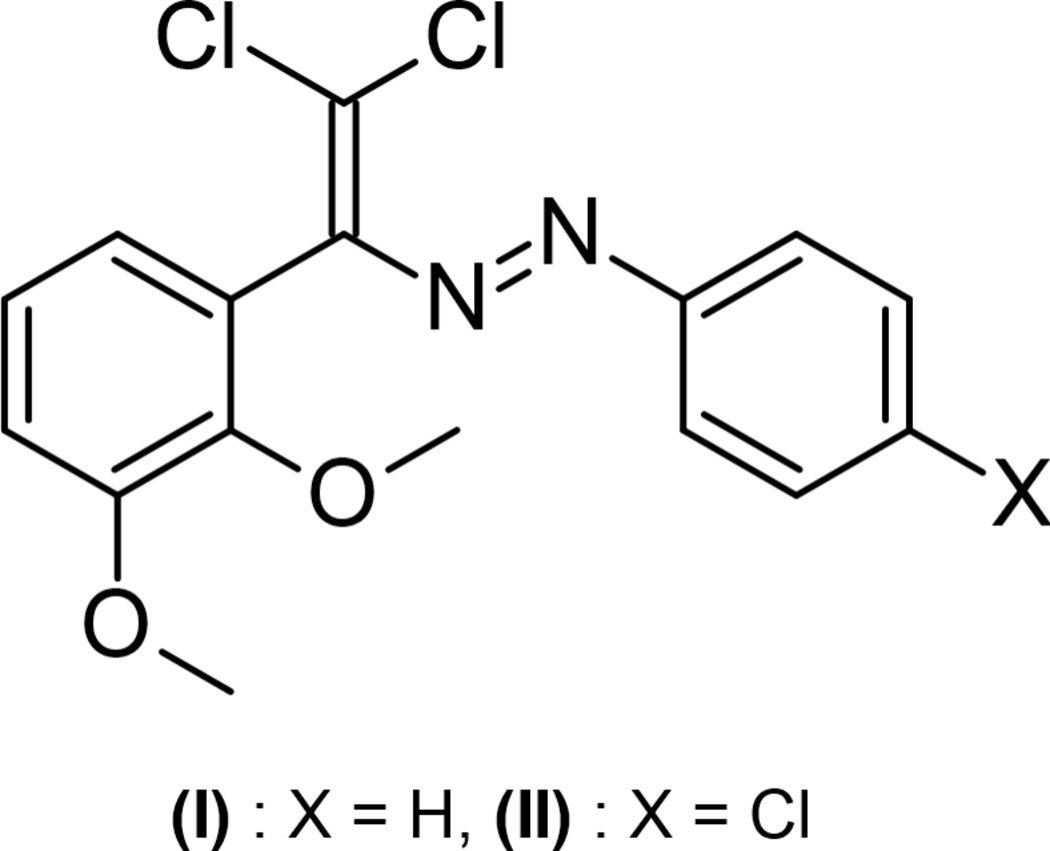


## 2. Structural commentary

**Figure 2:**
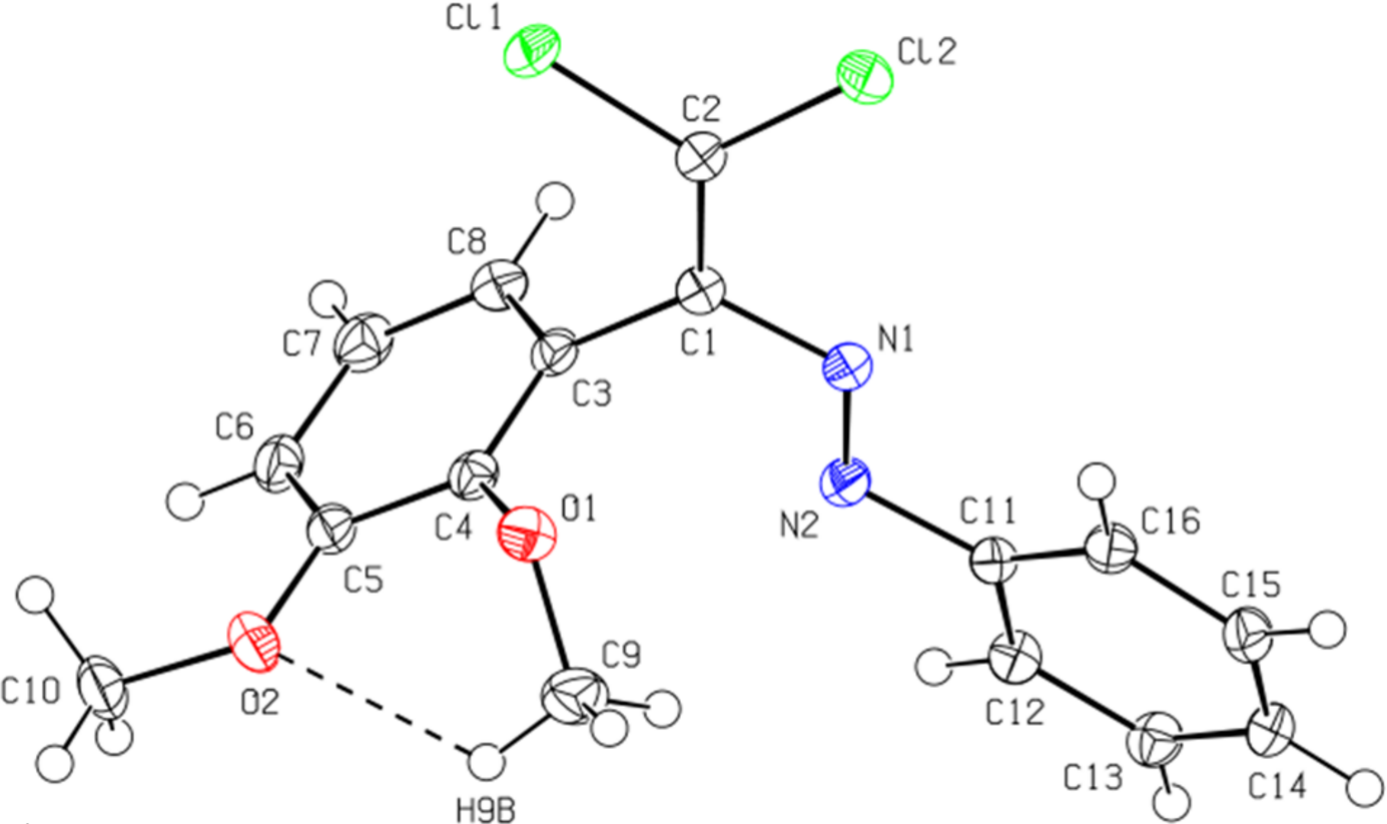
The mol­ecular structure of (**I**), showing the atom labelling and displacement ellipsoids drawn at the 50% probability level. The short contact is indicated by a dashed line.

The central mol­ecular fragment of (**I**), C1/C2/N1/N2/C3/C11/Cl1/Cl2, is almost planar (Fig. 2 ▸), with a root-mean-square (r.m.s.) deviation of fitted atoms from the least-squares plane of 0.0304 Å. This plane forms dihedral angles of 80.8 (1) and 26.7 (1) °, respectively, with the planes of the C3–C8 and C11–C16 benzene rings. The conformation of mol­ecule (**I**) may be consolidated by a short C—H⋯O contact (Table 1 ▸, Fig. 2 ▸), forming an *S*(6) motif.

**Figure 3:**
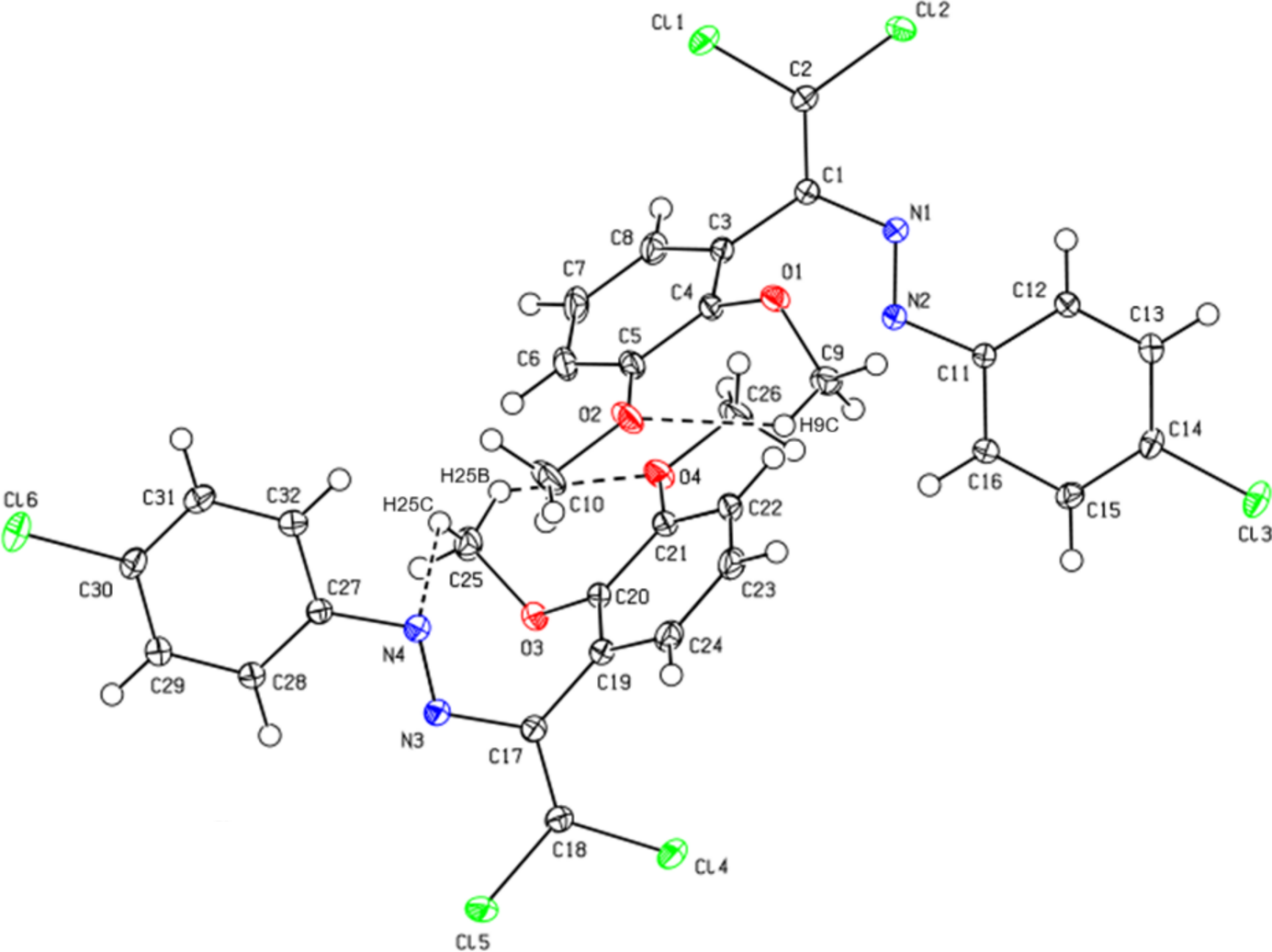
The mol­ecular structure of (**II**), showing the atom labelling and displacement ellipsoids drawn at the 50% probability level. Hydrogen bonds are indicated by dashed lines.

**Figure 4:**
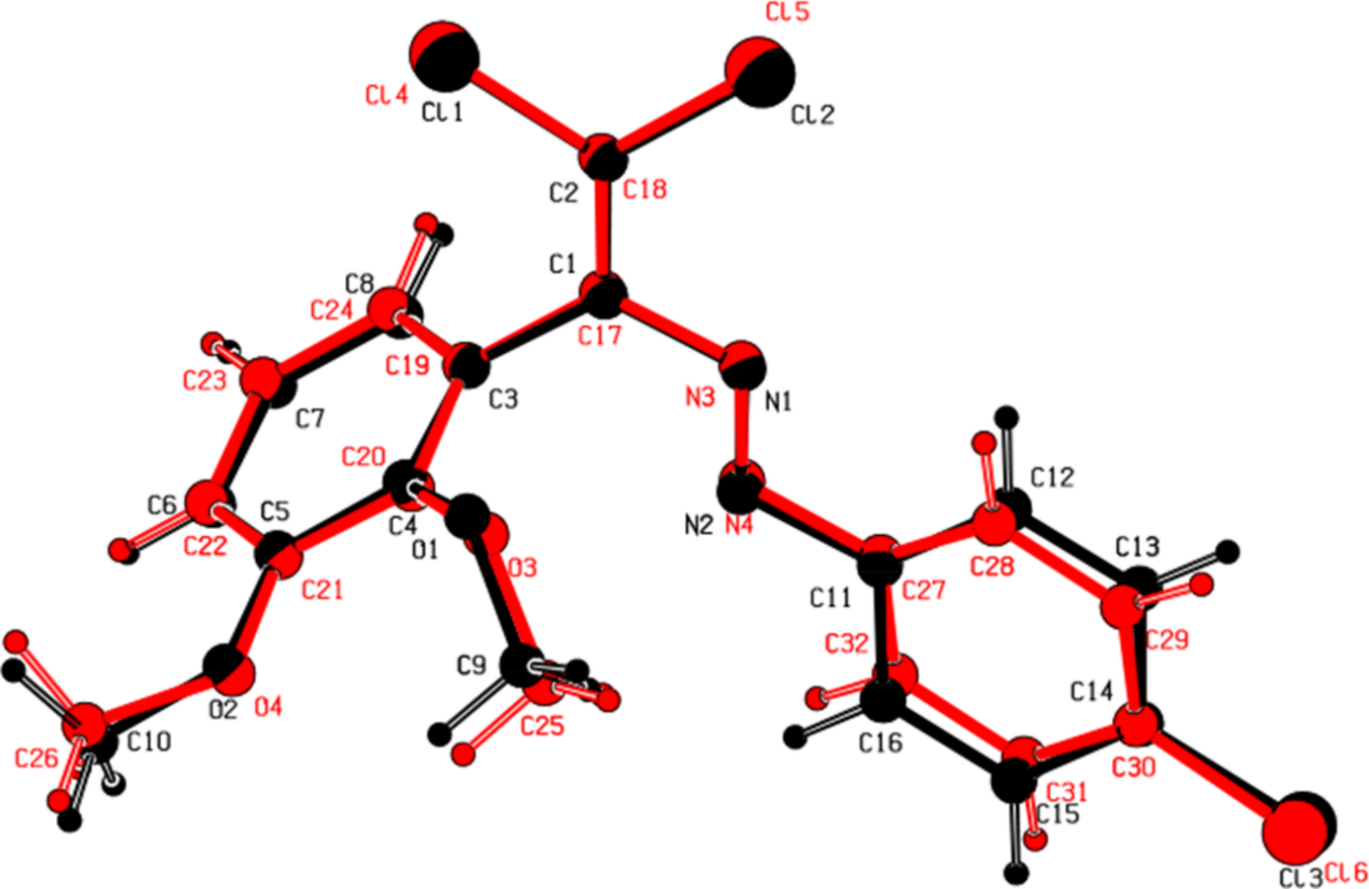
A least-squares overlay of the two independent mol­ecules *A* and *B* of (II) [inverted mol­ecule *B* (red) on mol­ecule *A* (black)].

There are two symmetry-independent mol­ecules, *A* (containing N1) and *B* (containing N3), in the asymmetric unit of (**II**) (Fig. 3 ▸). An overlay fit of inverted mol­ecule *B* on mol­ecule *A* is shown in Fig. 4 ▸, the weighted r.m.s. fit of the 17 non-H atoms being 0.200 Å and showing the differences to be in the chloro­phenyl groups C11–C16 and C27–C32. The central mol­ecular fragment of mol­ecule *A*, C1/C2/N2/N1/C3/C11/Cl1/Cl2, is also close to planar with an r.m.s. deviation of fitted atoms of 0.0226 Å (Fig. 3 ▸) and makes dihedral angles of 72.9 (1) and 6.6 (1)°, respectively, with the planes of the C3–C8 and C11–C16 benzene rings. The central mol­ecular fragment of mol­ecule *B*, C17/C18/N3/N4/C19/C27/Cl4/Cl5, is likewise almost planar with an r.m.s. deviation of fitted atoms of 0.0472 Å (Fig. 3 ▸) and makes dihedral angles of 69.1 (1) and 22.7 (1)°, respectively, with the planes of the (C19–C24) and (C27–C32) benzene rings. The conformation of mol­ecule *A* features an intra­molecular C—H⋯O hydrogen bond forming an *S*(6) motif, while the conformation of mol­ecule *B* features intra­molecular C—H⋯O and C—H⋯N hydrogen bonds (Table 2 ▸), which form *S*(6) and *S*(8) motifs, respectively.

## 3. Supra­molecular features and Hirshfeld surface analyses

**Figure 5:**
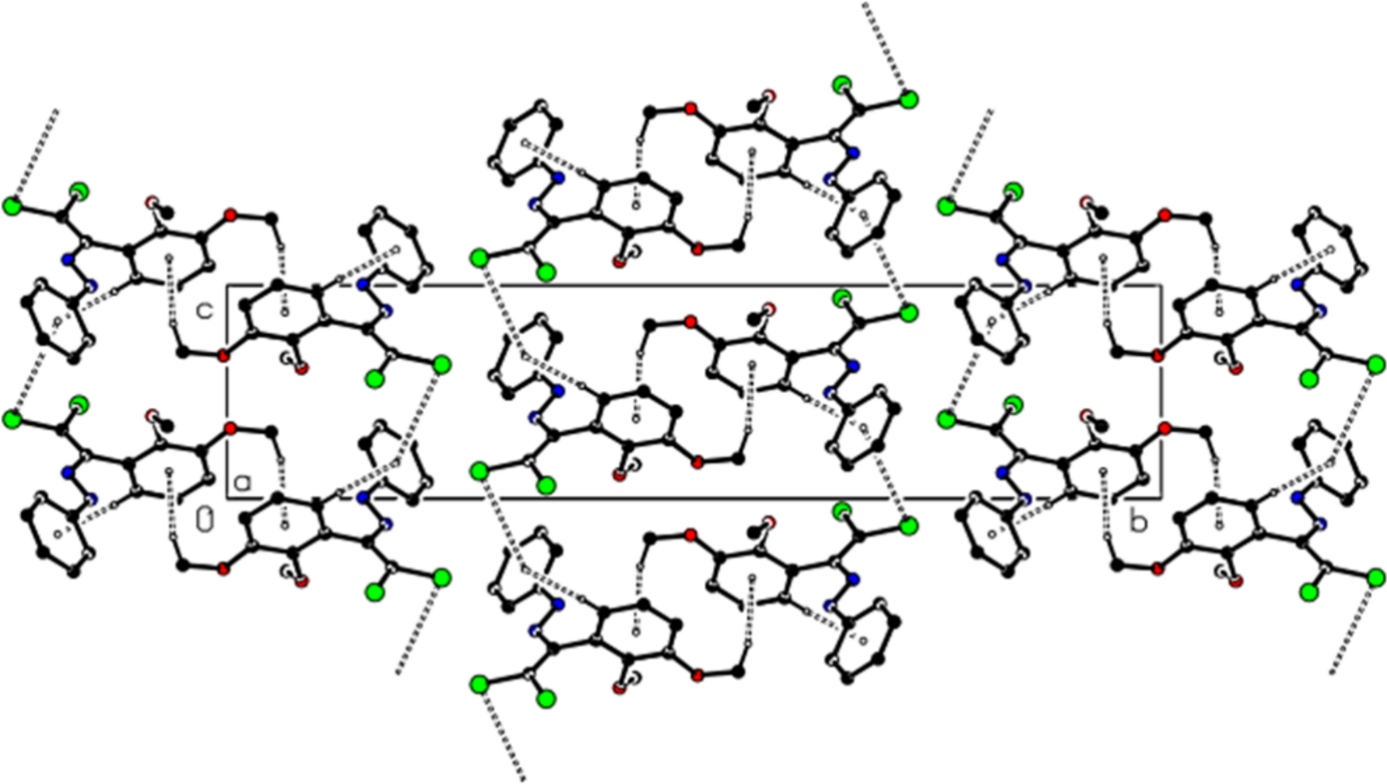
View of the C—H⋯π and C—Cl⋯π inter­actions of (**I**) along the *a* axis. H atoms not involved in hydrogen bonding were removed for clarity.

In the crystal structure of (**I**), the mol­ecules form layers parallel to the (020) plane through C—H⋯π and C—Cl⋯π inter­actions [C2—Cl2⋯*Cg*2^*a*^: C2—Cl2 = 1.7131 (15) Å, Cl2⋯*Cg*2^*a*^ = 3.9882 (7) Å, C2⋯*Cg*2^*a*^ = 4.2031 (16) Å, C2—Cl2⋯*Cg*2^*a*^ = 85.07 (5)°, Symmetry code: (*a*) *x*, *y*, 1 + *z*; where *Cg*2 is the centroid of the (C11–C16) benzene ring] (Table 1 ▸, Figs. 5 ▸ and 6 ▸). van der Waals inter­actions between these layers consolidate the packing.

**Figure 7:**
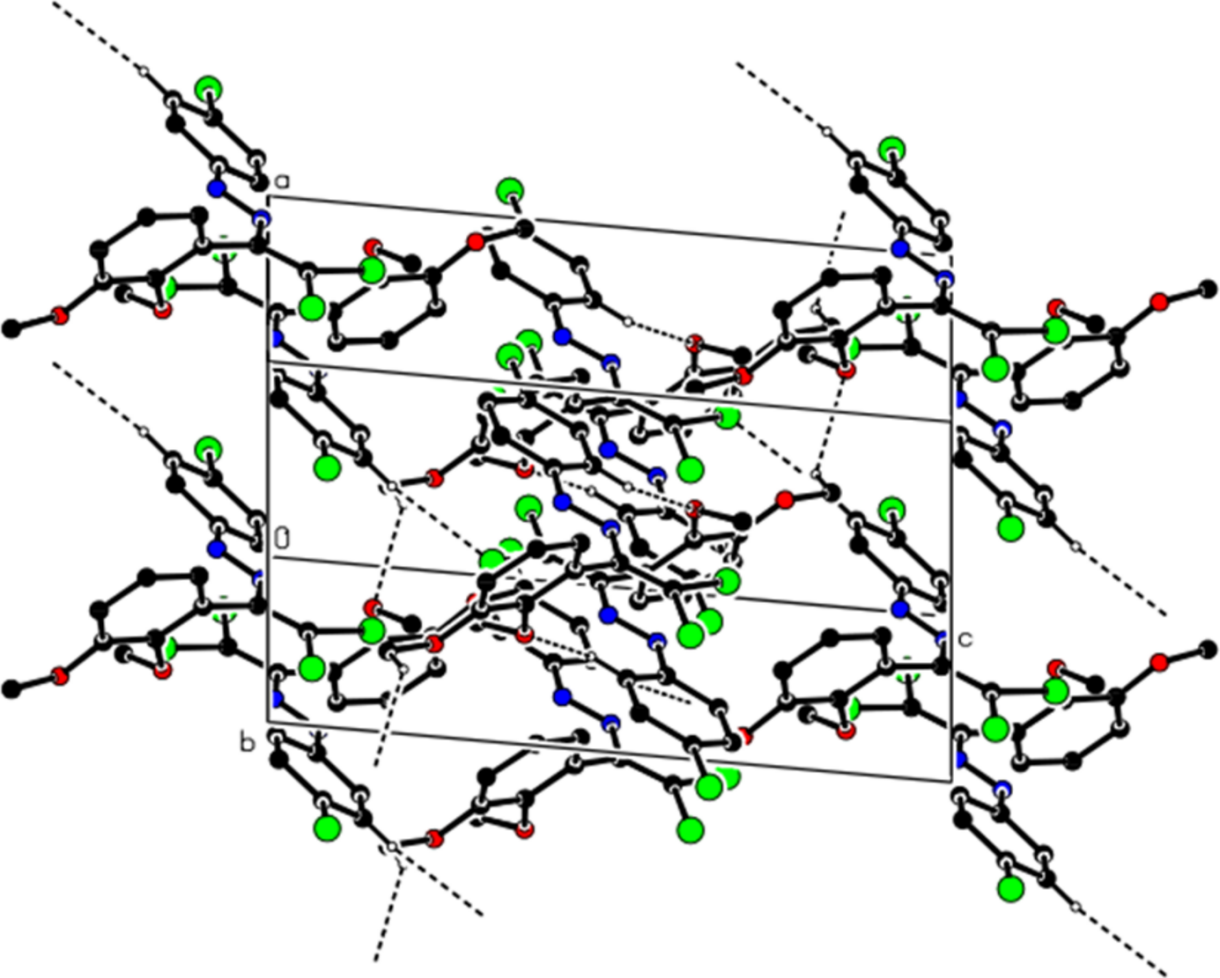
View of the C—H⋯O and C—H—Cl inter­actions (**II**) along the *b* axis. H atoms not involved in hydrogen bonding were removed for clarity.

In the crystal of (**II**), the mol­ecules are connected by C—H⋯O and C—H⋯Cl hydrogen bonds, forming a three-dimensional network (Table 2 ▸, Figs. 7 ▸ and 8 ▸). Additionally, C—Cl⋯π inter­actions [C2—Cl2⋯*Cg*2^*b*^: C2—Cl2 = 1.7149 (9) Å, Cl2⋯*Cg*2^*b*^ = 3.4334 (7) Å, C2⋯*Cg*2^*b*^ = 3.7761 (11) Å, C2—Cl2⋯*Cg*2^*b*^ = 87.72 (3), and C18—Cl5⋯*Cg*4^*c*^: C18—Cl5 = 1.7159 (9) Å, Cl5⋯*Cg*4^*c*^ = 3.8775 (7) Å, C18⋯*Cg*4^*c*^ = 4.1527 (13) Å, C18—Cl5⋯*Cg*4^*c*^ = 86.84 (4)°, Symmetry codes: (*b*) 1 − *x*, −*y*, 1 − *z*; (c) 2 − *x*, 1 − *y*, −*z*; where *Cg*2 and *Cg*4 are the centroids of the chloro­phenyl rings (C11–C16 and C27–C32) of mol­ecules *A* and *B*, respectively] also contribute to the packing.

**Figure 9:**
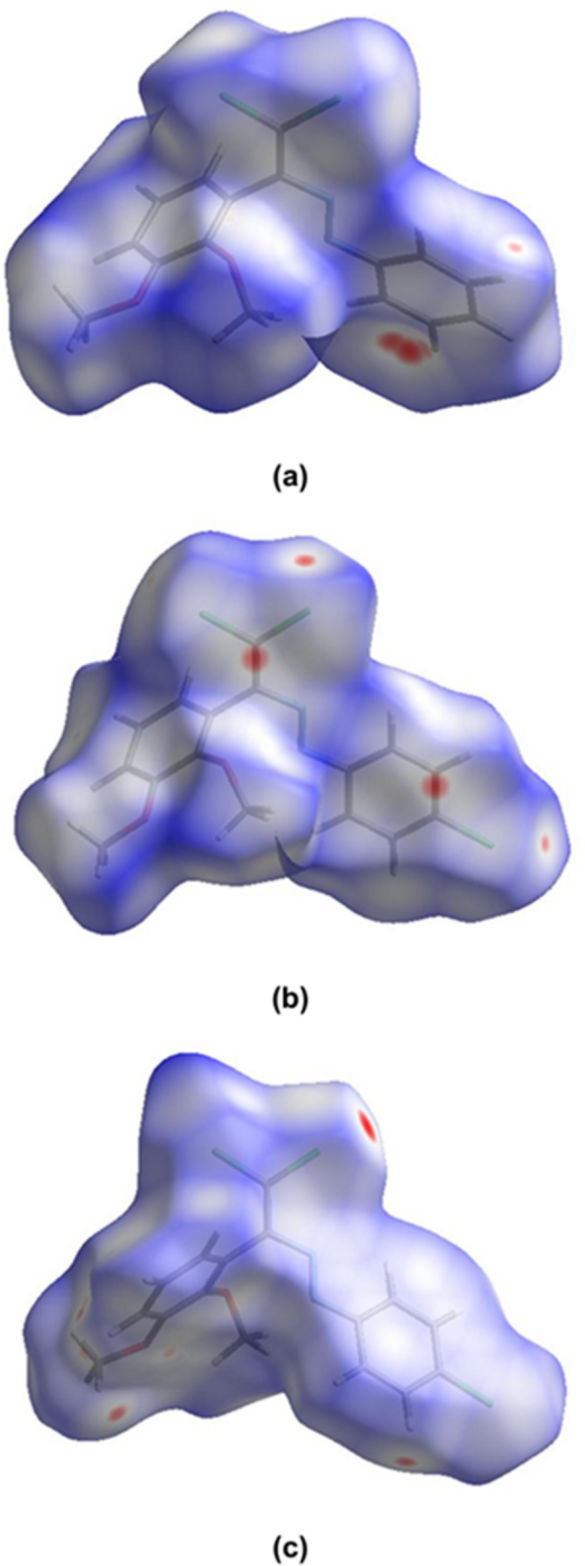
The Hirshfeld surfaces of (*a*) (**I**), (*b*) (**II**) mol­ecule *A* and (*c*) (**II**) mol­ecule *B* plotted over *d*_norm_.

**Figure 10:**
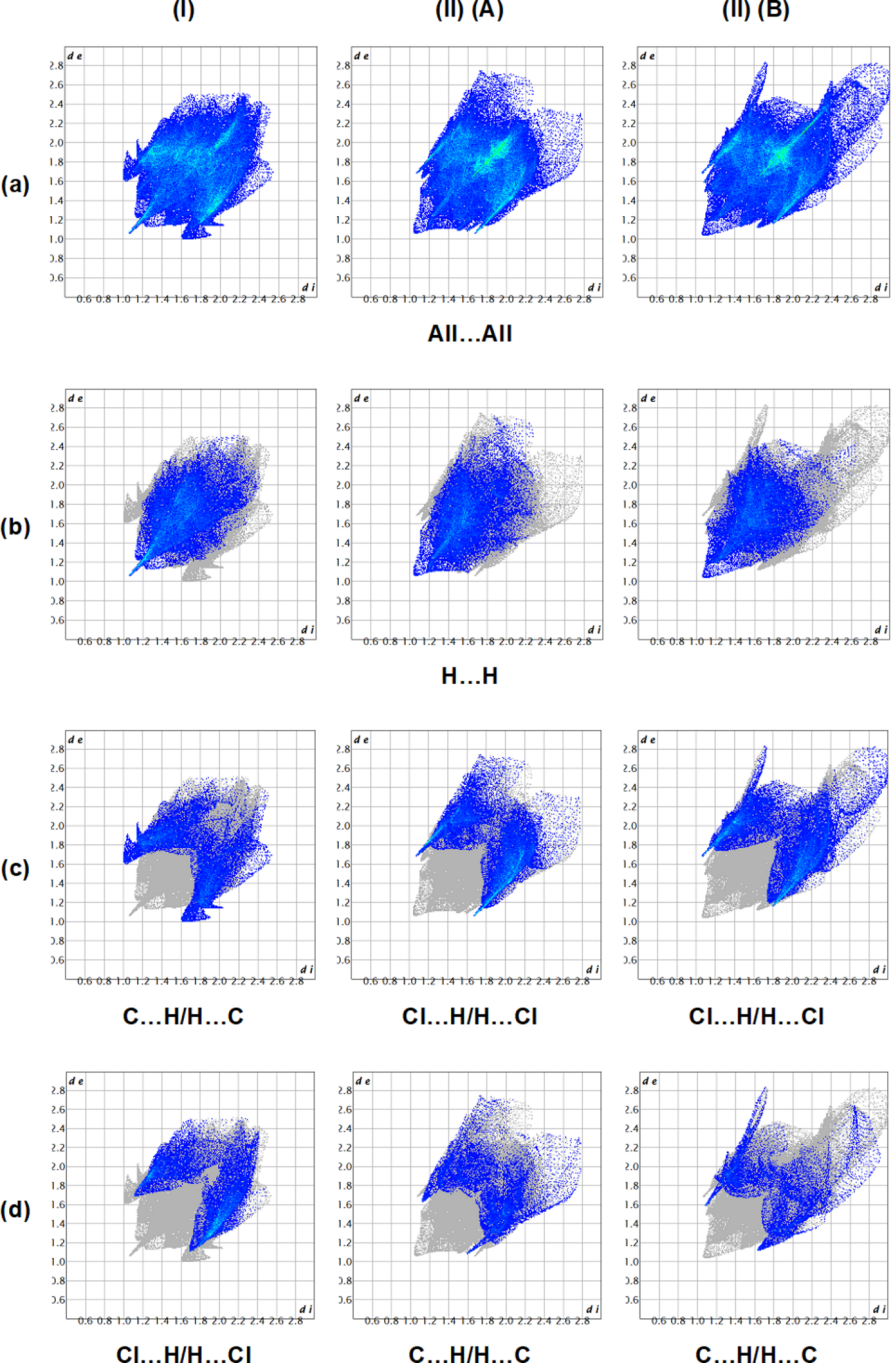
The full two-dimensional fingerprint plots for (**I**) and (**II**), showing (*a*) all inter­actions, and delineated into (*b*) H⋯H, (*c*) C⋯H/H⋯C for (**I**) [Cl⋯H/H⋯Cl for mol­ecules *A* and *B* of (**II**)] and (*d*) Cl⋯H/H⋯Cl for (**I**) (C⋯H/H⋯C for mol­ecules *A* and *B* of (**II**)] inter­actions. The *d*_i_ and *d*_e_ values are the closest inter­nal and external distances (in Å) from given points on the Hirshfeld surface.

*Crystal Explorer 17.5* (Spackman *et al.*, 2021 ▸) was used to generate Hirshfeld surfaces in the crystal structures of (**I**) and (**II**). The *d*_norm_ mappings for (**I**) and mol­ecules *A* and *B* of (**II**) were performed in the ranges −0.12 to 1.21 a.u., −0.10 to 1.35 a.u. and −0.10 to 1.66 a.u., respectively. The C⋯H/H⋯C, Cl⋯H/H⋯Cl and O⋯H/H⋯O inter­actions are indicated by red areas on the Hirshfeld surfaces (Fig. 9 ▸*a* for (**I**) and Fig. 9 ▸*c*,*d* for mol­ecules *A* and *B* of (**II**). The two-dimensional fingerprint plots are shown in Fig. 10 ▸. The dominant inter­actions in the crystal packing of the title compounds are H⋯H [(**I**): 35.9%, (**II**) *A*: 29.6% and (**II**) *B*: 28.2%], C⋯H/H⋯C [(**I**): 21.1%, (**II**) *A*: 13.6% and (**II**) *B*: 12.0%], Cl⋯H/H⋯Cl [(**I**): 20.2%, (**II**) *A*: 29.1% and (**II**) *B*: 31.3%], O⋯H/H⋯O [(**I**): 7.6%, (**II**) *A*: 7.5% and (**II**) *B*: 7.1%]. The presence of different functional groups in the compounds leads to some differences in the remaining weak inter­actions.

## 4. Database survey

A search of the Cambridge Structural Database (CSD, Version 6.00, update of April 2025; Groom *et al.*, 2016 ▸) for the (*E*)-1-(2,2-di­chloro-1-phenyl­ethen-1-yl)-2-phenyl­diazene moiety resulted in 39 hits. Eight compounds are most similar to the title compound, *viz*. those with CSD refcodes POCXIS (Shikhaliyev *et al.*, 2024 ▸), NIKXEO (Maharramov *et al.*, 2023 ▸), TAZDIL (Atioğlu *et al.*, 2022 ▸), HEHKEO (Akkurt *et al.*, 2022 ▸), PAXDOL (Çelikesir *et al.*, 2022 ▸), CANVUM (Shikhaliyev *et al.*, 2021 ▸), GUPHIL (Özkaraca *et al.*, 2020 ▸), HODQAV (Shikhaliyev *et al.*, 2019 ▸).

In the crystal of POCXIS, mol­ecules are linked by C—H⋯N hydrogen bonds, forming chains with *C*(6) motifs parallel to the *b* axis. Short inter­molecular Cl⋯O contacts of 2.8421 (16) Å and weak van der Waals inter­actions between these chains consolidate the crystal structure. In the crystal structure of NIKXEO, mol­ecules are linked by C—H⋯π and C—Cl⋯π inter­actions, forming layers parallel to (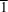
01). The cohesion of the packing is ensured by van der Waals forces between these layers. The mol­ecules in TAZDIL are joined into layers parallel to (011) by C—H⋯O and C—H⋯F hydrogen bonds. C—Br⋯π and C—F⋯π contacts, as well as π–π stacking inter­actions consolidate the crystal packing. C—H⋯Br inter­actions connect the mol­ecules in the crystal of the polymorph-1 of HEHKEO, resulting in zigzag *C*(8) chains parallel to [100]. These chains are connected by C—Br⋯π inter­actions into layers parallel to (001). van der Waals inter­actions between the layers contribute to the crystal cohesion. The mol­ecules in the crystal of PAXDOL are connected into chains running parallel to [001] by C—H⋯O hydrogen bonds. C—F⋯π contacts and π–π stacking inter­actions help to consolidate the crystal packing, and short Br⋯O [2.9828 (13) Å] distances are also observed. In CANVUM, the mol­ecules are linked by C—H⋯N inter­actions along [100], forming a *C*(6) chain. The mol­ecules are further connected by C—Cl⋯π inter­actions and face-to-face π–π stacking inter­actions, resulting in ribbons along [100]. In GUPHIL, mol­ecules are associated into inversion dimers *via* short Cl⋯Cl contacts [3.3763 (9) Å]. In HODQAV, mol­ecules are stacked in columns along [100] *via* weak C—H⋯Cl hydrogen bonds and face-to-face π–π stacking inter­actions. The crystal packing is further consolidated by short Cl⋯Cl contacts.

## 5. Synthesis and crystallization

Compounds (**I**) and (**II**) were synthesized according to a literature protocol (Shikhaliyev *et al.*, 2018 ▸). For (**I**), a 20 ml screw-neck vial was charged with dimethylsulfoxide (DMSO) (10 ml), (*E*)-1-(4-chloro­phen­yl)-2-(2,3-di­meth­oxy­benzyl­idene)hydrazine (290 mg, 1 mmol), tetra­methyl­ethylenedi­amine (TMEDA) (295 mg, 2.5 mmol), CuCl (2 mg, 0.02 mmol) and CCl_4_ (1 mmol). After 2–3 h (until TLC analysis showed complete consumption of the corresponding Schiff base), the reaction mixture was poured into a 0.01 *M* solution of HCl (100 ml, pH = 2–3), and extracted with di­chloro­methane (3 × 20 ml). The combined organic phase was washed with water (3 × 50 ml), brine (30 ml), dried over anhydrous Na_2_SO_4_ and concentrated in vacuum using a rotary evaporator. The residue was purified by column chromatography on silica gel using appropriate mixtures of hexane and di­chloro­methane (*v*/*v*: 5/1–3/1–1/1). A red solid was obtained (yield 65%); m.p. 365 K. ^1^H NMR (300 MHz, chloro­form-*d*) δ 7.71–7.69 (*m*, 2H, arom), 7.49–7.45 (*m*, 2H, arom), 7.30–7.25 (*m*, 1H, arom), 7.19–7.11 (*m*, 3H, arom), 3.71 (*s*, 3H, –OCH_3_), 3.84 (*s*, 3H, OCH_3_). ^13^C NMR (75 MHz, CDCl_3_) 152.4, 151.7, 149.5, 133.8, 130.7, 129.2, 128.3, 128.1, 127.7, 124.9, 121.7, 117.6, 60.6, 56.0.

For (**II**), the procedure was the same as that for (**I**) using methyl (*E*)-1-(4-chloro­phen­yl)-2-(2,3-di­meth­oxy­benzyl­idene)hydrazine (290 mg, 1 mmol). A red solid was obtained (yield 78%); m.p. 399 K. ^1^H NMR (300 MHz, chloro­form-*d*) δ 7.67–7.60 (*m*, 1H, arom), 7.52–7.45 (*m*, 1H, arom), 7.18–7.05 (*m*, 1H, arom), 3.75 (*s*, 1H, –OCH_3_), 3.98 (*s*, 1H, –OCH_3_). ^13^C NMR (75 MHz, CDCl_3_) 151.8, 151.0, 149.7, 136.7, 133.3, 129.1, 128.4, 128.0, 127.6, 124.6, 117.7, 114.6, 67.6, 54.6.

In each case, the obtained compound was dissolved in di­chloro­methane and then left at room temperature for slow evaporation; red single crystals suitable for X-ray diffraction analysis started to form after *ca* 2 d.

## 6. Refinement

Crystal data, data collection and structure refinement details are summarized in Table 3 ▸. H atoms were positioned geometrically and refined using a riding model [C—H = 0.95–0.98 Å and *U*_iso_(H) = 1.2 or 1.5 *U*_eq_(C)].

## Supplementary Material

Crystal structure: contains datablock(s) I, II. DOI: 10.1107/S2056989026000137/nx2031sup1.cif

Structure factors: contains datablock(s) I. DOI: 10.1107/S2056989026000137/nx2031Isup2.hkl

Structure factors: contains datablock(s) II. DOI: 10.1107/S2056989026000137/nx2031IIsup3.hkl

CCDC references: 2521000, 2520999

Additional supporting information:  crystallographic information; 3D view; checkCIF report

## Figures and Tables

**Figure 6:**
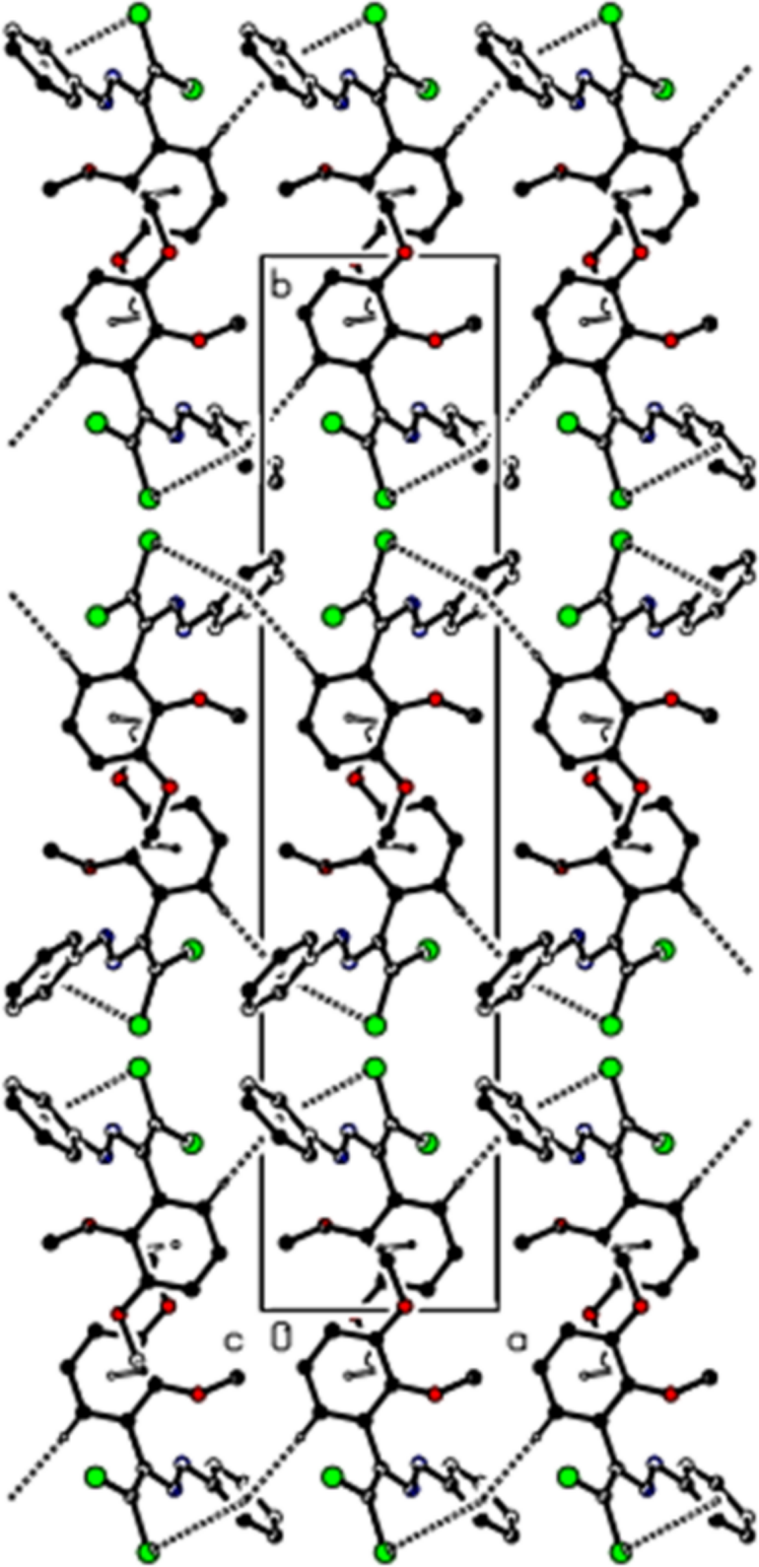
View of the C—H⋯π and C—Cl⋯π inter­actions of (**I**) along the *c* axis. H atoms not involved in hydrogen bonding were removed for clarity.

**Figure 8:**
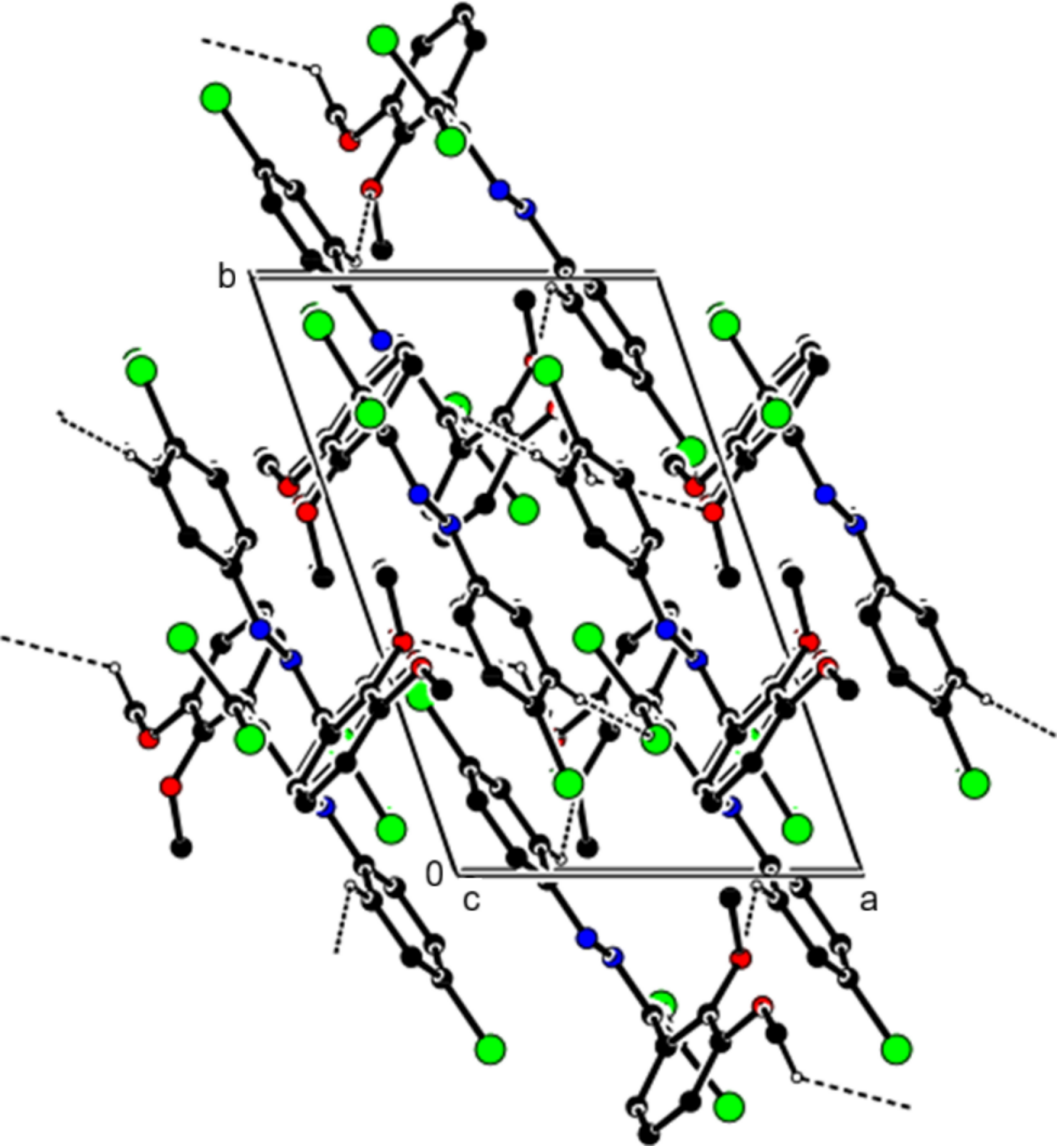
View of the C—H⋯O and C—H—Cl inter­actions (**II**) along the *c* axis. H atoms not involved in hydrogen bonding were removed for clarity.

**Table 1:** Hydrogen-bond geometry (Å, °) for (**I**) *Cg* and *Cg*2 are the centroids of the C3–C8 and C11–C16 rings, respectively.

*D*—H⋯*A*	*D*—H	H⋯*A*	*D*⋯*A*	*D*—H⋯*A*
C9—H9*B*⋯O2	0.98	2.35	2.955 (2)	119
C8—H8⋯*Cg*2^i^	0.95	2.52	3.4665 (16)	174
C10—H10*A*⋯*Cg*1^ii^	0.98	2.77	3.6231 (18)	146

Symmetry codes: (i) 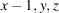; (ii) 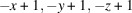.

**Table 2:** Hydrogen-bond geometry (Å, °) for (**II**)

*D*—H⋯*A*	*D*—H	H⋯*A*	*D*⋯*A*	*D*—H⋯*A*
C9—H9*C*⋯O2	0.98	2.32	2.9059 (12)	117
C10—H10*C*⋯O3^i^	0.98	2.65	3.3241 (13)	126
C12—H12⋯O1^ii^	0.95	2.66	3.2919 (11)	125
C25—H25*B*⋯O4	0.98	2.33	2.9226 (12)	118
C25—H25*C*⋯N4	0.98	2.58	3.2210 (13)	124
C31—H31⋯Cl2^iii^	0.95	2.87	3.7991 (10)	166

Symmetry codes: (i) 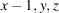; (ii) 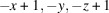; (iii) 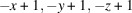.

**Table 3:** Experimental details

	(**I**)	(**II**)
Crystal data		
Chemical formula	C_16_H_14_Cl_2_N_2_O_2_	C_16_H_13_Cl_3_N_2_O_2_
*M*_r_	337.19	371.63
Crystal system, space group	Monoclinic, *P*2_1_/*c*	Triclinic, *P* 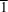
Temperature (K)	100	100
*a*, *b*, *c* (Å)	7.30389 (4), 30.68147 (13), 7.42658 (4)	8.2181 (11), 13.3651 (17), 16.687 (2)
α, β, γ (°)	90, 109.7560 (6), 90	108.139 (3), 94.732 (3), 106.396 (3)
*V* (Å^3^)	1566.30 (2)	1641.5 (4)
*Z*	4	4
Radiation type	Cu *K*α	Mo *K*α
μ (mm^−1^)	3.80	0.57
Crystal size (mm)	0.13 × 0.11 × 0.06	0.30 × 0.20 × 0.20

Data collection		
Diffractometer	Rigaku XtaLAB Synergy-S, HyPix-6000HE area-detector	Bruker D8 QUEST PHOTON-III area detector
Absorption correction	Gaussian (*CrysAlis PRO*; Rigaku OD, 2025 ▸)	Multi-scan (*SADABS*; Krause *et al.*, 2015 ▸)
*T*_min_, *T*_max_	0.641, 0.796	0.656, 0.746
No. of measured, independent and observed [*I* > 2σ(*I*)] reflections	81705, 3424, 3376	39419, 11888, 10714
*R*_int_	0.046	0.023
(sin θ/λ)_max_ (Å^−1^)	0.639	0.758

Refinement		
*R*[*F*^2^ > 2σ(*F*^2^)], *wR*(*F*^2^), *S*	0.032, 0.082, 1.06	0.027, 0.074, 1.05
No. of reflections	3424	11888
No. of parameters	201	419
H-atom treatment	H-atom parameters constrained	H-atom parameters constrained
Δρ_max_, Δρ_min_ (e Å^−3^)	0.33, −0.36	0.48, −0.36

Computer programs: *CrysAlis PRO* (Rigaku OD, 2021 ▸), *APEX3* and *SAINT* (Bruker, 2018 ▸), *SHELXT2014/5* (Sheldrick, 2015*a* ▸), *SHELXL2018/3* (Sheldrick, 2015*b* ▸), *ORTEP-3 for Windows* (Farrugia, 2012 ▸) and *PLATON* (Spek, 2020 ▸).

## supplementary crystallographic information

### (E)-1-[2,2-Dichloro-1-(2,3-dimethoxyphenyl)ethen-1-yl]-2-phenyldiazene (I) Crystal data

**Table:**

C_16_H_14_Cl_2_N_2_O_2_	*F*(000) = 696
*M**_r_* = 337.19	*D*_x_ = 1.430 Mg m^−^^3^
Monoclinic, *P*2_1_/*c*	Cu *K*α radiation, λ = 1.54184 Å
*a* = 7.30389 (4) Å	Cell parameters from 60750 reflections
*b* = 30.68147 (13) Å	θ = 2.9–79.6°
*c* = 7.42658 (4) Å	µ = 3.80 mm^−^^1^
β = 109.7560 (6)°	*T* = 100 K
*V* = 1566.30 (2) Å^3^	Prism, brown
*Z* = 4	0.13 × 0.11 × 0.06 mm

### (E)-1-[2,2-Dichloro-1-(2,3-dimethoxyphenyl)ethen-1-yl]-2-phenyldiazene (I) Data collection

**Table:**

Rigaku XtaLAB Synergy-S, HyPix-6000HE area-detector diffractometer	3376 reflections with *I* > 2σ(*I*)
Radiation source: micro-focus sealed X-ray tube	*R*_int_ = 0.046
φ and ω scans	θ_max_ = 80.0°, θ_min_ = 2.9°
Absorption correction: gaussian (CrysAlisPro; Rigaku OD, 2025)	*h* = −9→9
*T*_min_ = 0.641, *T*_max_ = 0.796	*k* = −39→39
81705 measured reflections	*l* = −9→9
3424 independent reflections	

### (E)-1-[2,2-Dichloro-1-(2,3-dimethoxyphenyl)ethen-1-yl]-2-phenyldiazene (I) Refinement

**Table:**

Refinement on *F*^2^	Primary atom site location: difference Fourier map
Least-squares matrix: full	Secondary atom site location: difference Fourier map
*R*[*F*^2^ > 2σ(*F*^2^)] = 0.032	Hydrogen site location: inferred from neighbouring sites
*wR*(*F*^2^) = 0.082	H-atom parameters constrained
*S* = 1.06	*w* = 1/[σ^2^(*F*_o_^2^) + (0.0377*P*)^2^ + 1.1051*P*] where *P* = (*F*_o_^2^ + 2*F*_c_^2^)/3
3424 reflections	(Δ/σ)_max_ = 0.001
201 parameters	Δρ_max_ = 0.33 e Å^−^^3^
0 restraints	Δρ_min_ = −0.36 e Å^−^^3^

### (E)-1-[2,2-Dichloro-1-(2,3-dimethoxyphenyl)ethen-1-yl]-2-phenyldiazene (I) Special details

**Table:**

Geometry. All esds (except the esd in the dihedral angle between two l.s. planes) are estimated using the full covariance matrix. The cell esds are taken into account individually in the estimation of esds in distances, angles and torsion angles; correlations between esds in cell parameters are only used when they are defined by crystal symmetry. An approximate (isotropic) treatment of cell esds is used for estimating esds involving l.s. planes.

### (E)-1-[2,2-Dichloro-1-(2,3-dimethoxyphenyl)ethen-1-yl]-2-phenyldiazene (I) Fractional atomic coordinates and isotropic or equivalent isotropic displacement parameters (Å^2^)

**Table:**

	*x*	*y*	*z*	*U*_iso_*/*U*_eq_	
Cl1	0.29682 (5)	0.65828 (2)	0.93930 (5)	0.02571 (10)	
Cl2	0.52050 (5)	0.72971 (2)	0.87184 (5)	0.02478 (10)	
O1	0.73197 (15)	0.57980 (3)	0.88689 (15)	0.0228 (2)	
O2	0.60438 (16)	0.49625 (3)	0.82871 (15)	0.0249 (2)	
N1	0.63222 (17)	0.66977 (4)	0.62019 (17)	0.0194 (2)	
N2	0.66515 (17)	0.64600 (4)	0.49630 (17)	0.0193 (2)	
C1	0.5041 (2)	0.65062 (4)	0.7039 (2)	0.0186 (3)	
C2	0.4487 (2)	0.67660 (5)	0.8224 (2)	0.0205 (3)	
C3	0.4334 (2)	0.60489 (4)	0.6629 (2)	0.0183 (3)	
C4	0.5525 (2)	0.57056 (4)	0.75418 (19)	0.0185 (3)	
C5	0.4827 (2)	0.52744 (5)	0.7230 (2)	0.0201 (3)	
C6	0.2983 (2)	0.51953 (5)	0.5918 (2)	0.0232 (3)	
H6	0.251670	0.490476	0.566572	0.028*	
C7	0.1820 (2)	0.55417 (5)	0.4975 (2)	0.0255 (3)	
H7	0.056650	0.548542	0.407235	0.031*	
C8	0.2470 (2)	0.59666 (5)	0.5339 (2)	0.0233 (3)	
H8	0.165342	0.620149	0.471536	0.028*	
C9	0.8957 (2)	0.56394 (6)	0.8398 (3)	0.0316 (4)	
H9A	0.895488	0.577293	0.719845	0.047*	
H9B	0.886635	0.532197	0.824871	0.047*	
H9C	1.016507	0.571543	0.942622	0.047*	
C10	0.5289 (3)	0.45272 (5)	0.8119 (2)	0.0284 (3)	
H10A	0.502301	0.442238	0.680886	0.043*	
H10B	0.408232	0.452573	0.842048	0.043*	
H10C	0.624663	0.433599	0.901379	0.043*	
C11	0.7989 (2)	0.66527 (4)	0.4167 (2)	0.0184 (3)	
C12	0.7998 (2)	0.64778 (5)	0.2436 (2)	0.0202 (3)	
H12	0.713531	0.624722	0.184687	0.024*	
C13	0.9273 (2)	0.66424 (5)	0.1579 (2)	0.0222 (3)	
H13	0.925250	0.653168	0.037768	0.027*	
C14	1.0577 (2)	0.69685 (5)	0.2478 (2)	0.0226 (3)	
H14	1.146914	0.707599	0.190471	0.027*	
C15	1.0584 (2)	0.71388 (5)	0.4217 (2)	0.0230 (3)	
H15	1.148738	0.736065	0.482808	0.028*	
C16	0.9280 (2)	0.69868 (4)	0.5064 (2)	0.0206 (3)	
H16	0.926334	0.710772	0.623627	0.025*	

### (E)-1-[2,2-Dichloro-1-(2,3-dimethoxyphenyl)ethen-1-yl]-2-phenyldiazene (I) Atomic displacement parameters (Å^2^)

**Table:**

	*U*^11^	*U*^22^	*U*^33^	*U*^12^	*U*^13^	*U*^23^
Cl1	0.02833 (18)	0.02687 (18)	0.02930 (19)	−0.00008 (13)	0.01938 (15)	−0.00127 (13)
Cl2	0.03005 (19)	0.01812 (17)	0.02945 (19)	−0.00041 (12)	0.01436 (15)	−0.00447 (12)
O1	0.0207 (5)	0.0220 (5)	0.0233 (5)	−0.0005 (4)	0.0046 (4)	−0.0033 (4)
O2	0.0309 (5)	0.0166 (5)	0.0272 (5)	−0.0004 (4)	0.0098 (4)	0.0016 (4)
N1	0.0206 (5)	0.0185 (5)	0.0218 (6)	0.0010 (4)	0.0105 (5)	0.0007 (4)
N2	0.0191 (5)	0.0197 (5)	0.0206 (6)	−0.0003 (4)	0.0088 (5)	−0.0007 (4)
C1	0.0184 (6)	0.0182 (6)	0.0203 (7)	0.0006 (5)	0.0079 (5)	0.0007 (5)
C2	0.0211 (6)	0.0202 (6)	0.0225 (7)	0.0005 (5)	0.0103 (5)	0.0006 (5)
C3	0.0199 (6)	0.0191 (6)	0.0195 (6)	−0.0015 (5)	0.0114 (5)	−0.0014 (5)
C4	0.0201 (6)	0.0203 (6)	0.0175 (6)	−0.0019 (5)	0.0095 (5)	−0.0029 (5)
C5	0.0248 (7)	0.0186 (6)	0.0208 (7)	−0.0003 (5)	0.0128 (6)	−0.0005 (5)
C6	0.0253 (7)	0.0211 (7)	0.0271 (7)	−0.0065 (5)	0.0138 (6)	−0.0044 (6)
C7	0.0194 (7)	0.0290 (8)	0.0280 (7)	−0.0046 (6)	0.0079 (6)	−0.0040 (6)
C8	0.0192 (6)	0.0240 (7)	0.0278 (7)	0.0014 (5)	0.0093 (6)	0.0005 (6)
C9	0.0206 (7)	0.0295 (8)	0.0432 (10)	0.0007 (6)	0.0090 (7)	−0.0034 (7)
C10	0.0393 (9)	0.0158 (7)	0.0332 (8)	−0.0017 (6)	0.0163 (7)	0.0016 (6)
C11	0.0194 (6)	0.0172 (6)	0.0204 (6)	0.0017 (5)	0.0091 (5)	0.0018 (5)
C12	0.0208 (6)	0.0208 (6)	0.0185 (6)	−0.0012 (5)	0.0061 (5)	−0.0015 (5)
C13	0.0251 (7)	0.0252 (7)	0.0178 (7)	0.0006 (6)	0.0093 (6)	0.0000 (5)
C14	0.0248 (7)	0.0217 (7)	0.0258 (7)	0.0000 (5)	0.0144 (6)	0.0035 (6)
C15	0.0258 (7)	0.0181 (6)	0.0277 (7)	−0.0033 (5)	0.0124 (6)	−0.0018 (6)
C16	0.0239 (7)	0.0188 (6)	0.0214 (7)	−0.0008 (5)	0.0107 (6)	−0.0029 (5)

### (E)-1-[2,2-Dichloro-1-(2,3-dimethoxyphenyl)ethen-1-yl]-2-phenyldiazene (I) Geometric parameters (Å, º)

**Table:**

Cl1—C2	1.7182 (14)	C8—H8	0.9500
Cl2—C2	1.7131 (15)	C9—H9A	0.9800
O1—C4	1.3772 (17)	C9—H9B	0.9800
O1—C9	1.4395 (19)	C9—H9C	0.9800
O2—C5	1.3604 (18)	C10—H10A	0.9800
O2—C10	1.4341 (17)	C10—H10B	0.9800
N1—N2	1.2593 (17)	C10—H10C	0.9800
N1—C1	1.4141 (18)	C11—C12	1.3950 (19)
N2—C11	1.4296 (18)	C11—C16	1.3993 (19)
C1—C2	1.347 (2)	C12—C13	1.389 (2)
C1—C3	1.4905 (19)	C12—H12	0.9500
C3—C4	1.388 (2)	C13—C14	1.387 (2)
C3—C8	1.398 (2)	C13—H13	0.9500
C4—C5	1.4086 (19)	C14—C15	1.391 (2)
C5—C6	1.391 (2)	C14—H14	0.9500
C6—C7	1.393 (2)	C15—C16	1.388 (2)
C6—H6	0.9500	C15—H15	0.9500
C7—C8	1.382 (2)	C16—H16	0.9500
C7—H7	0.9500		
			
C4—O1—C9	115.13 (11)	O1—C9—H9B	109.5
C5—O2—C10	116.62 (12)	H9A—C9—H9B	109.5
N2—N1—C1	113.49 (12)	O1—C9—H9C	109.5
N1—N2—C11	112.86 (11)	H9A—C9—H9C	109.5
C2—C1—N1	115.51 (12)	H9B—C9—H9C	109.5
C2—C1—C3	122.04 (13)	O2—C10—H10A	109.5
N1—C1—C3	122.44 (12)	O2—C10—H10B	109.5
C1—C2—Cl2	124.42 (11)	H10A—C10—H10B	109.5
C1—C2—Cl1	121.64 (11)	O2—C10—H10C	109.5
Cl2—C2—Cl1	113.94 (8)	H10A—C10—H10C	109.5
C4—C3—C8	120.14 (13)	H10B—C10—H10C	109.5
C4—C3—C1	119.85 (12)	C12—C11—C16	120.56 (13)
C8—C3—C1	120.01 (13)	C12—C11—N2	115.99 (12)
O1—C4—C3	118.77 (12)	C16—C11—N2	123.41 (12)
O1—C4—C5	120.99 (13)	C13—C12—C11	119.68 (13)
C3—C4—C5	120.01 (13)	C13—C12—H12	120.2
O2—C5—C6	124.78 (13)	C11—C12—H12	120.2
O2—C5—C4	115.87 (13)	C14—C13—C12	119.95 (13)
C6—C5—C4	119.34 (13)	C14—C13—H13	120.0
C5—C6—C7	120.04 (13)	C12—C13—H13	120.0
C5—C6—H6	120.0	C13—C14—C15	120.28 (13)
C7—C6—H6	120.0	C13—C14—H14	119.9
C8—C7—C6	120.71 (14)	C15—C14—H14	119.9
C8—C7—H7	119.6	C16—C15—C14	120.46 (13)
C6—C7—H7	119.6	C16—C15—H15	119.8
C7—C8—C3	119.66 (14)	C14—C15—H15	119.8
C7—C8—H8	120.2	C15—C16—C11	119.04 (13)
C3—C8—H8	120.2	C15—C16—H16	120.5
O1—C9—H9A	109.5	C11—C16—H16	120.5
			
C1—N1—N2—C11	−178.40 (11)	C3—C4—C5—O2	−175.49 (12)
N2—N1—C1—C2	−173.35 (13)	O1—C4—C5—C6	178.10 (12)
N2—N1—C1—C3	6.15 (19)	C3—C4—C5—C6	3.7 (2)
N1—C1—C2—Cl2	0.89 (19)	O2—C5—C6—C7	176.99 (13)
C3—C1—C2—Cl2	−178.62 (11)	C4—C5—C6—C7	−2.1 (2)
N1—C1—C2—Cl1	−178.88 (10)	C5—C6—C7—C8	−0.6 (2)
C3—C1—C2—Cl1	1.6 (2)	C6—C7—C8—C3	1.7 (2)
C2—C1—C3—C4	−101.19 (16)	C4—C3—C8—C7	−0.1 (2)
N1—C1—C3—C4	79.34 (17)	C1—C3—C8—C7	−179.60 (13)
C2—C1—C3—C8	78.30 (18)	N1—N2—C11—C12	−161.90 (12)
N1—C1—C3—C8	−101.17 (16)	N1—N2—C11—C16	20.25 (19)
C9—O1—C4—C3	−117.64 (14)	C16—C11—C12—C13	−1.2 (2)
C9—O1—C4—C5	67.90 (17)	N2—C11—C12—C13	−179.13 (13)
C8—C3—C4—O1	−177.11 (12)	C11—C12—C13—C14	2.3 (2)
C1—C3—C4—O1	2.38 (19)	C12—C13—C14—C15	−1.5 (2)
C8—C3—C4—C5	−2.6 (2)	C13—C14—C15—C16	−0.4 (2)
C1—C3—C4—C5	176.89 (12)	C14—C15—C16—C11	1.5 (2)
C10—O2—C5—C6	−4.7 (2)	C12—C11—C16—C15	−0.7 (2)
C10—O2—C5—C4	174.42 (12)	N2—C11—C16—C15	177.09 (13)
O1—C4—C5—O2	−1.09 (19)		

### (E)-1-[2,2-Dichloro-1-(2,3-dimethoxyphenyl)ethen-1-yl]-2-phenyldiazene (I) Hydrogen-bond geometry (Å, º)

*Cg* and *Cg*2 are the centroids of the C3–C8 and C11–C16 rings, respectively.

**Table:**

*D*—H···*A*	*D*—H	H···*A*	*D*···*A*	*D*—H···*A*
C9—H9*B*···O2	0.98	2.35	2.955 (2)	119
C8—H8···*Cg*2^i^	0.95	2.52	3.4665 (16)	174
C10—H10*A*···*Cg*1^ii^	0.98	2.77	3.6231 (18)	146

Symmetry codes: (i) *x*−1, *y*, *z*; (ii) −*x*+1, −*y*+1, −*z*+1.

### (E)-1-(4-Chlorophenyl)-2-[2,2-dichloro-1-(2,3-dimethoxyphenyl)ethen-1-yl]diazene (II) Crystal data

**Table:**

C_16_H_13_Cl_3_N_2_O_2_	*Z* = 4
*M**_r_* = 371.63	*F*(000) = 760
Triclinic, *P*1	*D*_x_ = 1.504 Mg m^−^^3^
*a* = 8.2181 (11) Å	Mo *K*α radiation, λ = 0.71073 Å
*b* = 13.3651 (17) Å	Cell parameters from 9797 reflections
*c* = 16.687 (2) Å	θ = 2.5–32.6°
α = 108.139 (3)°	µ = 0.57 mm^−^^1^
β = 94.732 (3)°	*T* = 100 K
γ = 106.396 (3)°	Prism, red
*V* = 1641.5 (4) Å^3^	0.30 × 0.20 × 0.20 mm

### (E)-1-(4-Chlorophenyl)-2-[2,2-dichloro-1-(2,3-dimethoxyphenyl)ethen-1-yl]diazene (II) Data collection

**Table:**

Bruker D8 QUEST PHOTON-III area detector diffractometer	10714 reflections with *I* > 2σ(*I*)
Radiation source: fine-focus sealed X-ray tube	*R*_int_ = 0.023
φ and ω scans	θ_max_ = 32.6°, θ_min_ = 2.5°
Absorption correction: multi-scan (SADABS; Krause *et al.*, 2015)	*h* = −12→12
*T*_min_ = 0.656, *T*_max_ = 0.746	*k* = −20→20
39419 measured reflections	*l* = −25→25
11888 independent reflections	

### (E)-1-(4-Chlorophenyl)-2-[2,2-dichloro-1-(2,3-dimethoxyphenyl)ethen-1-yl]diazene (II) Refinement

**Table:**

Refinement on *F*^2^	Primary atom site location: difference Fourier map
Least-squares matrix: full	Secondary atom site location: difference Fourier map
*R*[*F*^2^ > 2σ(*F*^2^)] = 0.027	Hydrogen site location: inferred from neighbouring sites
*wR*(*F*^2^) = 0.074	H-atom parameters constrained
*S* = 1.05	*w* = 1/[σ^2^(*F*_o_^2^) + (0.0326*P*)^2^ + 0.5553*P*] where *P* = (*F*_o_^2^ + 2*F*_c_^2^)/3
11888 reflections	(Δ/σ)_max_ = 0.002
419 parameters	Δρ_max_ = 0.48 e Å^−^^3^
0 restraints	Δρ_min_ = −0.36 e Å^−^^3^

### (E)-1-(4-Chlorophenyl)-2-[2,2-dichloro-1-(2,3-dimethoxyphenyl)ethen-1-yl]diazene (II) Special details

**Table:**

Geometry. All esds (except the esd in the dihedral angle between two l.s. planes) are estimated using the full covariance matrix. The cell esds are taken into account individually in the estimation of esds in distances, angles and torsion angles; correlations between esds in cell parameters are only used when they are defined by crystal symmetry. An approximate (isotropic) treatment of cell esds is used for estimating esds involving l.s. planes.

### (E)-1-(4-Chlorophenyl)-2-[2,2-dichloro-1-(2,3-dimethoxyphenyl)ethen-1-yl]diazene (II) Fractional atomic coordinates and isotropic or equivalent isotropic displacement parameters (Å^2^)

**Table:**

	*x*	*y*	*z*	*U*_iso_*/*U*_eq_	
Cl1	0.52462 (3)	0.39382 (2)	0.61838 (2)	0.01884 (4)	
Cl2	0.60551 (3)	0.22480 (2)	0.67158 (2)	0.01681 (4)	
Cl3	0.93613 (3)	−0.29765 (2)	0.35426 (2)	0.02135 (5)	
O1	0.37126 (8)	0.14223 (5)	0.37547 (4)	0.01618 (11)	
O2	0.35903 (9)	0.22142 (7)	0.24322 (4)	0.02241 (14)	
N1	0.68510 (9)	0.14189 (6)	0.50182 (5)	0.01367 (12)	
N2	0.73277 (9)	0.10900 (6)	0.43084 (5)	0.01397 (12)	
C1	0.63666 (10)	0.23844 (7)	0.51515 (5)	0.01289 (13)	
C2	0.59557 (10)	0.28070 (7)	0.59243 (5)	0.01407 (13)	
C3	0.62956 (11)	0.28996 (7)	0.44848 (5)	0.01406 (13)	
C4	0.49846 (11)	0.23529 (7)	0.37639 (5)	0.01431 (14)	
C5	0.49164 (12)	0.28101 (8)	0.31110 (6)	0.01749 (15)	
C6	0.61659 (13)	0.38110 (8)	0.31980 (7)	0.02166 (17)	
H6	0.613973	0.411847	0.275667	0.026*	
C7	0.74526 (13)	0.43615 (8)	0.39303 (7)	0.02283 (18)	
H7	0.828671	0.504994	0.398958	0.027*	
C8	0.75279 (12)	0.39143 (7)	0.45739 (6)	0.01894 (16)	
H8	0.840912	0.429363	0.507151	0.023*	
C9	0.34619 (13)	0.03990 (8)	0.30589 (6)	0.02063 (16)	
H9A	0.266099	−0.022038	0.316964	0.031*	
H9B	0.457253	0.027063	0.301213	0.031*	
H9C	0.298159	0.045034	0.252128	0.031*	
C10	0.34774 (15)	0.26519 (11)	0.17551 (7)	0.0288 (2)	
H10A	0.247916	0.215008	0.130545	0.043*	
H10B	0.453296	0.271712	0.151302	0.043*	
H10C	0.334288	0.338809	0.198379	0.043*	
C11	0.78013 (10)	0.01095 (7)	0.41738 (5)	0.01290 (13)	
C12	0.77031 (10)	−0.04677 (7)	0.47494 (5)	0.01369 (13)	
H12	0.730021	−0.021214	0.526810	0.016*	
C13	0.81964 (11)	−0.14140 (7)	0.45591 (6)	0.01522 (14)	
H13	0.814229	−0.180789	0.494720	0.018*	
C14	0.87724 (11)	−0.17801 (7)	0.37917 (6)	0.01531 (14)	
C15	0.88686 (11)	−0.12229 (7)	0.32107 (6)	0.01720 (15)	
H15	0.925946	−0.148619	0.268961	0.021*	
C16	0.83798 (12)	−0.02699 (7)	0.34081 (6)	0.01643 (14)	
H16	0.844063	0.012369	0.301954	0.020*	
Cl4	0.87637 (4)	0.07775 (2)	−0.06471 (2)	0.02627 (5)	
Cl5	0.82442 (3)	0.22427 (2)	−0.15224 (2)	0.02417 (5)	
Cl6	0.64871 (3)	0.84635 (2)	0.08704 (2)	0.02619 (5)	
O3	1.06237 (8)	0.39299 (5)	0.15401 (4)	0.01643 (11)	
O4	1.08535 (9)	0.35191 (6)	0.30450 (4)	0.02088 (13)	
N3	0.77232 (10)	0.36141 (6)	0.00963 (5)	0.01578 (13)	
N4	0.72217 (10)	0.41370 (6)	0.07466 (5)	0.01555 (13)	
C17	0.80095 (11)	0.26384 (7)	0.01445 (5)	0.01528 (14)	
C18	0.82960 (12)	0.19689 (8)	−0.05837 (6)	0.01803 (15)	
C19	0.80494 (11)	0.23840 (7)	0.09495 (5)	0.01545 (14)	
C20	0.93752 (11)	0.30709 (7)	0.16454 (5)	0.01428 (14)	
C21	0.94769 (12)	0.28288 (7)	0.24077 (5)	0.01667 (15)	
C22	0.81979 (13)	0.19287 (8)	0.24688 (6)	0.02044 (17)	
H22	0.823347	0.177453	0.298738	0.025*	
C23	0.68692 (13)	0.12549 (8)	0.17728 (7)	0.02210 (17)	
H23	0.600506	0.064214	0.182018	0.027*	
C24	0.67918 (12)	0.14677 (8)	0.10112 (6)	0.01977 (16)	
H24	0.589309	0.099575	0.053525	0.024*	
C25	1.07785 (14)	0.50288 (8)	0.21075 (6)	0.02244 (17)	
H25A	1.155871	0.558731	0.192716	0.034*	
H25B	1.124324	0.512107	0.269563	0.034*	
H25C	0.963877	0.512497	0.208544	0.034*	
C26	1.11744 (14)	0.31627 (10)	0.37516 (6)	0.02507 (19)	
H26A	1.226263	0.367450	0.413125	0.038*	
H26B	1.125226	0.241010	0.353401	0.038*	
H26C	1.022764	0.315986	0.407247	0.038*	
C27	0.70345 (11)	0.51546 (7)	0.07168 (5)	0.01464 (14)	
C28	0.76452 (12)	0.56438 (7)	0.01249 (6)	0.01693 (15)	
H28	0.818802	0.528373	−0.030412	0.020*	
C29	0.74563 (12)	0.66564 (8)	0.01657 (6)	0.01828 (15)	
H29	0.786462	0.699392	−0.023503	0.022*	
C30	0.66612 (12)	0.71729 (7)	0.08007 (6)	0.01769 (15)	
C31	0.60443 (13)	0.66986 (8)	0.13918 (6)	0.02090 (17)	
H31	0.549572	0.705915	0.181751	0.025*	
C32	0.62434 (12)	0.56855 (8)	0.13499 (6)	0.01901 (16)	
H32	0.583913	0.535295	0.175387	0.023*	

### (E)-1-(4-Chlorophenyl)-2-[2,2-dichloro-1-(2,3-dimethoxyphenyl)ethen-1-yl]diazene (II) Atomic displacement parameters (Å^2^)

**Table:**

	*U*^11^	*U*^22^	*U*^33^	*U*^12^	*U*^13^	*U*^23^
Cl1	0.02007 (9)	0.01638 (9)	0.02093 (9)	0.00975 (7)	0.00648 (7)	0.00359 (7)
Cl2	0.01901 (9)	0.02017 (9)	0.01194 (8)	0.00688 (7)	0.00406 (6)	0.00578 (7)
Cl3	0.02032 (9)	0.01497 (9)	0.03167 (11)	0.00974 (7)	0.00761 (8)	0.00779 (8)
O1	0.0172 (3)	0.0169 (3)	0.0139 (3)	0.0034 (2)	0.0043 (2)	0.0062 (2)
O2	0.0260 (3)	0.0327 (4)	0.0171 (3)	0.0146 (3)	0.0051 (2)	0.0154 (3)
N1	0.0155 (3)	0.0132 (3)	0.0131 (3)	0.0058 (2)	0.0034 (2)	0.0045 (2)
N2	0.0166 (3)	0.0132 (3)	0.0140 (3)	0.0064 (2)	0.0046 (2)	0.0055 (2)
C1	0.0138 (3)	0.0122 (3)	0.0130 (3)	0.0046 (3)	0.0029 (2)	0.0044 (3)
C2	0.0141 (3)	0.0136 (3)	0.0146 (3)	0.0056 (3)	0.0032 (3)	0.0038 (3)
C3	0.0164 (3)	0.0136 (3)	0.0158 (3)	0.0074 (3)	0.0060 (3)	0.0070 (3)
C4	0.0164 (3)	0.0155 (3)	0.0153 (3)	0.0077 (3)	0.0063 (3)	0.0081 (3)
C5	0.0209 (4)	0.0230 (4)	0.0178 (4)	0.0137 (3)	0.0087 (3)	0.0124 (3)
C6	0.0275 (4)	0.0234 (4)	0.0274 (4)	0.0155 (4)	0.0144 (4)	0.0181 (4)
C7	0.0256 (4)	0.0167 (4)	0.0328 (5)	0.0083 (3)	0.0123 (4)	0.0147 (4)
C8	0.0196 (4)	0.0144 (4)	0.0240 (4)	0.0053 (3)	0.0066 (3)	0.0078 (3)
C9	0.0261 (4)	0.0180 (4)	0.0150 (4)	0.0042 (3)	0.0031 (3)	0.0049 (3)
C10	0.0344 (5)	0.0474 (6)	0.0228 (4)	0.0253 (5)	0.0113 (4)	0.0245 (5)
C11	0.0150 (3)	0.0122 (3)	0.0131 (3)	0.0056 (3)	0.0041 (2)	0.0052 (3)
C12	0.0151 (3)	0.0139 (3)	0.0134 (3)	0.0051 (3)	0.0038 (3)	0.0059 (3)
C13	0.0151 (3)	0.0143 (3)	0.0177 (4)	0.0050 (3)	0.0029 (3)	0.0074 (3)
C14	0.0143 (3)	0.0119 (3)	0.0207 (4)	0.0056 (3)	0.0042 (3)	0.0056 (3)
C15	0.0199 (4)	0.0161 (4)	0.0176 (4)	0.0079 (3)	0.0078 (3)	0.0057 (3)
C16	0.0218 (4)	0.0159 (4)	0.0150 (3)	0.0083 (3)	0.0076 (3)	0.0070 (3)
Cl4	0.04214 (14)	0.02113 (10)	0.01987 (10)	0.01977 (10)	0.00606 (9)	0.00440 (8)
Cl5	0.03724 (12)	0.02648 (11)	0.01195 (9)	0.01681 (9)	0.00405 (8)	0.00530 (8)
Cl6	0.02955 (11)	0.01739 (10)	0.03471 (13)	0.01311 (9)	0.00505 (9)	0.00860 (9)
O3	0.0186 (3)	0.0153 (3)	0.0166 (3)	0.0047 (2)	0.0048 (2)	0.0075 (2)
O4	0.0265 (3)	0.0255 (3)	0.0147 (3)	0.0114 (3)	0.0034 (2)	0.0101 (2)
N3	0.0188 (3)	0.0153 (3)	0.0143 (3)	0.0077 (3)	0.0029 (2)	0.0049 (2)
N4	0.0173 (3)	0.0153 (3)	0.0157 (3)	0.0072 (2)	0.0038 (2)	0.0056 (2)
C17	0.0176 (3)	0.0142 (3)	0.0144 (3)	0.0065 (3)	0.0025 (3)	0.0045 (3)
C18	0.0236 (4)	0.0175 (4)	0.0139 (3)	0.0100 (3)	0.0023 (3)	0.0040 (3)
C19	0.0197 (4)	0.0142 (3)	0.0152 (3)	0.0080 (3)	0.0057 (3)	0.0061 (3)
C20	0.0181 (3)	0.0139 (3)	0.0145 (3)	0.0079 (3)	0.0058 (3)	0.0068 (3)
C21	0.0231 (4)	0.0182 (4)	0.0148 (3)	0.0119 (3)	0.0073 (3)	0.0083 (3)
C22	0.0304 (4)	0.0193 (4)	0.0213 (4)	0.0140 (3)	0.0140 (3)	0.0126 (3)
C23	0.0288 (4)	0.0151 (4)	0.0275 (4)	0.0087 (3)	0.0143 (4)	0.0106 (3)
C24	0.0226 (4)	0.0139 (4)	0.0225 (4)	0.0055 (3)	0.0069 (3)	0.0056 (3)
C25	0.0292 (5)	0.0150 (4)	0.0197 (4)	0.0048 (3)	−0.0016 (3)	0.0051 (3)
C26	0.0324 (5)	0.0386 (6)	0.0165 (4)	0.0223 (4)	0.0088 (3)	0.0153 (4)
C27	0.0163 (3)	0.0149 (3)	0.0133 (3)	0.0064 (3)	0.0026 (3)	0.0046 (3)
C28	0.0215 (4)	0.0174 (4)	0.0153 (3)	0.0095 (3)	0.0062 (3)	0.0067 (3)
C29	0.0226 (4)	0.0176 (4)	0.0177 (4)	0.0089 (3)	0.0047 (3)	0.0079 (3)
C30	0.0187 (4)	0.0143 (3)	0.0201 (4)	0.0077 (3)	0.0013 (3)	0.0044 (3)
C31	0.0247 (4)	0.0196 (4)	0.0210 (4)	0.0116 (3)	0.0088 (3)	0.0055 (3)
C32	0.0240 (4)	0.0192 (4)	0.0172 (4)	0.0101 (3)	0.0087 (3)	0.0069 (3)

### (E)-1-(4-Chlorophenyl)-2-[2,2-dichloro-1-(2,3-dimethoxyphenyl)ethen-1-yl]diazene (II) Geometric parameters (Å, º)

**Table:**

Cl1—C2	1.7161 (9)	Cl4—C18	1.7151 (9)
Cl2—C2	1.7149 (9)	Cl5—C18	1.7159 (9)
Cl3—C14	1.7379 (9)	Cl6—C30	1.7397 (9)
O1—C4	1.3753 (10)	O3—C20	1.3755 (10)
O1—C9	1.4401 (11)	O3—C25	1.4418 (12)
O2—C5	1.3601 (12)	O4—C21	1.3650 (12)
O2—C10	1.4325 (12)	O4—C26	1.4340 (11)
N1—N2	1.2646 (10)	N3—N4	1.2649 (10)
N1—C1	1.4139 (11)	N3—C17	1.4134 (11)
N2—C11	1.4277 (11)	N4—C27	1.4263 (11)
C1—C2	1.3498 (11)	C17—C18	1.3497 (12)
C1—C3	1.4846 (11)	C17—C19	1.4848 (12)
C3—C4	1.3915 (12)	C19—C20	1.3933 (12)
C3—C8	1.4004 (12)	C19—C24	1.4000 (12)
C4—C5	1.4096 (12)	C20—C21	1.4084 (12)
C5—C6	1.3943 (14)	C21—C22	1.3938 (13)
C6—C7	1.3930 (15)	C22—C23	1.3916 (15)
C6—H6	0.9500	C22—H22	0.9500
C7—C8	1.3869 (13)	C23—C24	1.3863 (14)
C7—H7	0.9500	C23—H23	0.9500
C8—H8	0.9500	C24—H24	0.9500
C9—H9A	0.9800	C25—H25A	0.9800
C9—H9B	0.9800	C25—H25B	0.9800
C9—H9C	0.9800	C25—H25C	0.9800
C10—H10A	0.9800	C26—H26A	0.9800
C10—H10B	0.9800	C26—H26B	0.9800
C10—H10C	0.9800	C26—H26C	0.9800
C11—C16	1.3959 (11)	C27—C32	1.3961 (12)
C11—C12	1.4012 (11)	C27—C28	1.3978 (12)
C12—C13	1.3875 (12)	C28—C29	1.3868 (12)
C12—H12	0.9500	C28—H28	0.9500
C13—C14	1.3942 (12)	C29—C30	1.3929 (13)
C13—H13	0.9500	C29—H29	0.9500
C14—C15	1.3891 (12)	C30—C31	1.3863 (13)
C15—C16	1.3934 (12)	C31—C32	1.3904 (13)
C15—H15	0.9500	C31—H31	0.9500
C16—H16	0.9500	C32—H32	0.9500
			
C4—O1—C9	117.08 (7)	C20—O3—C25	115.29 (7)
C5—O2—C10	117.18 (8)	C21—O4—C26	116.79 (8)
N2—N1—C1	113.70 (7)	N4—N3—C17	113.56 (7)
N1—N2—C11	113.47 (7)	N3—N4—C27	113.24 (7)
C2—C1—N1	115.71 (7)	C18—C17—N3	115.51 (8)
C2—C1—C3	121.82 (7)	C18—C17—C19	122.16 (8)
N1—C1—C3	122.46 (7)	N3—C17—C19	122.30 (7)
C1—C2—Cl2	123.13 (7)	C17—C18—Cl4	122.85 (7)
C1—C2—Cl1	122.29 (7)	C17—C18—Cl5	122.94 (7)
Cl2—C2—Cl1	114.57 (5)	Cl4—C18—Cl5	114.21 (5)
C4—C3—C8	120.33 (8)	C20—C19—C24	120.27 (8)
C4—C3—C1	118.81 (7)	C20—C19—C17	118.74 (8)
C8—C3—C1	120.86 (8)	C24—C19—C17	120.99 (8)
O1—C4—C3	117.55 (7)	O3—C20—C19	118.06 (7)
O1—C4—C5	122.34 (8)	O3—C20—C21	121.82 (8)
C3—C4—C5	119.88 (8)	C19—C20—C21	119.96 (8)
O2—C5—C6	125.08 (8)	O4—C21—C22	124.62 (8)
O2—C5—C4	115.52 (8)	O4—C21—C20	116.11 (8)
C6—C5—C4	119.40 (9)	C22—C21—C20	119.27 (8)
C7—C6—C5	120.18 (8)	C23—C22—C21	120.24 (8)
C7—C6—H6	119.9	C23—C22—H22	119.9
C5—C6—H6	119.9	C21—C22—H22	119.9
C8—C7—C6	120.65 (9)	C24—C23—C22	120.76 (9)
C8—C7—H7	119.7	C24—C23—H23	119.6
C6—C7—H7	119.7	C22—C23—H23	119.6
C7—C8—C3	119.53 (9)	C23—C24—C19	119.44 (9)
C7—C8—H8	120.2	C23—C24—H24	120.3
C3—C8—H8	120.2	C19—C24—H24	120.3
O1—C9—H9A	109.5	O3—C25—H25A	109.5
O1—C9—H9B	109.5	O3—C25—H25B	109.5
H9A—C9—H9B	109.5	H25A—C25—H25B	109.5
O1—C9—H9C	109.5	O3—C25—H25C	109.5
H9A—C9—H9C	109.5	H25A—C25—H25C	109.5
H9B—C9—H9C	109.5	H25B—C25—H25C	109.5
O2—C10—H10A	109.5	O4—C26—H26A	109.5
O2—C10—H10B	109.5	O4—C26—H26B	109.5
H10A—C10—H10B	109.5	H26A—C26—H26B	109.5
O2—C10—H10C	109.5	O4—C26—H26C	109.5
H10A—C10—H10C	109.5	H26A—C26—H26C	109.5
H10B—C10—H10C	109.5	H26B—C26—H26C	109.5
C16—C11—C12	120.20 (7)	C32—C27—C28	120.14 (8)
C16—C11—N2	115.60 (7)	C32—C27—N4	115.82 (8)
C12—C11—N2	124.20 (7)	C28—C27—N4	123.99 (8)
C13—C12—C11	119.76 (8)	C29—C28—C27	119.81 (8)
C13—C12—H12	120.1	C29—C28—H28	120.1
C11—C12—H12	120.1	C27—C28—H28	120.1
C12—C13—C14	119.25 (8)	C28—C29—C30	119.25 (8)
C12—C13—H13	120.4	C28—C29—H29	120.4
C14—C13—H13	120.4	C30—C29—H29	120.4
C15—C14—C13	121.82 (8)	C31—C30—C29	121.72 (8)
C15—C14—Cl3	118.73 (7)	C31—C30—Cl6	119.16 (7)
C13—C14—Cl3	119.44 (7)	C29—C30—Cl6	119.09 (7)
C14—C15—C16	118.62 (8)	C30—C31—C32	118.77 (8)
C14—C15—H15	120.7	C30—C31—H31	120.6
C16—C15—H15	120.7	C32—C31—H31	120.6
C15—C16—C11	120.35 (8)	C31—C32—C27	120.30 (8)
C15—C16—H16	119.8	C31—C32—H32	119.8
C11—C16—H16	119.8	C27—C32—H32	119.8
			
C1—N1—N2—C11	−179.49 (7)	C17—N3—N4—C27	176.47 (7)
N2—N1—C1—C2	−175.87 (7)	N4—N3—C17—C18	170.57 (8)
N2—N1—C1—C3	4.67 (11)	N4—N3—C17—C19	−11.33 (12)
N1—C1—C2—Cl2	1.60 (11)	N3—C17—C18—Cl4	177.08 (6)
C3—C1—C2—Cl2	−178.94 (6)	C19—C17—C18—Cl4	−1.02 (13)
N1—C1—C2—Cl1	−177.06 (6)	N3—C17—C18—Cl5	−2.27 (12)
C3—C1—C2—Cl1	2.41 (12)	C19—C17—C18—Cl5	179.62 (7)
C2—C1—C3—C4	−108.12 (10)	C18—C17—C19—C20	112.01 (10)
N1—C1—C3—C4	71.31 (10)	N3—C17—C19—C20	−65.97 (11)
C2—C1—C3—C8	72.24 (11)	C18—C17—C19—C24	−67.47 (12)
N1—C1—C3—C8	−108.33 (10)	N3—C17—C19—C24	114.55 (10)
C9—O1—C4—C3	−121.98 (8)	C25—O3—C20—C19	119.91 (8)
C9—O1—C4—C5	63.47 (11)	C25—O3—C20—C21	−64.64 (10)
C8—C3—C4—O1	−173.36 (8)	C24—C19—C20—O3	177.05 (8)
C1—C3—C4—O1	7.00 (11)	C17—C19—C20—O3	−2.43 (11)
C8—C3—C4—C5	1.34 (12)	C24—C19—C20—C21	1.52 (12)
C1—C3—C4—C5	−178.31 (7)	C17—C19—C20—C21	−177.96 (8)
C10—O2—C5—C6	0.14 (13)	C26—O4—C21—C22	12.60 (12)
C10—O2—C5—C4	−179.98 (8)	C26—O4—C21—C20	−167.81 (8)
O1—C4—C5—O2	−5.68 (12)	O3—C20—C21—O4	2.16 (12)
C3—C4—C5—O2	179.89 (7)	C19—C20—C21—O4	177.53 (8)
O1—C4—C5—C6	174.21 (8)	O3—C20—C21—C22	−178.23 (8)
C3—C4—C5—C6	−0.23 (12)	C19—C20—C21—C22	−2.87 (12)
O2—C5—C6—C7	178.86 (9)	O4—C21—C22—C23	−178.23 (8)
C4—C5—C6—C7	−1.01 (13)	C20—C21—C22—C23	2.20 (13)
C5—C6—C7—C8	1.15 (14)	C21—C22—C23—C24	−0.17 (14)
C6—C7—C8—C3	−0.04 (14)	C22—C23—C24—C19	−1.20 (14)
C4—C3—C8—C7	−1.20 (13)	C20—C19—C24—C23	0.52 (13)
C1—C3—C8—C7	178.43 (8)	C17—C19—C24—C23	179.99 (8)
N1—N2—C11—C16	−178.41 (7)	N3—N4—C27—C32	170.83 (8)
N1—N2—C11—C12	2.04 (12)	N3—N4—C27—C28	−11.65 (12)
C16—C11—C12—C13	0.52 (12)	C32—C27—C28—C29	−0.28 (13)
N2—C11—C12—C13	−179.95 (8)	N4—C27—C28—C29	−177.70 (8)
C11—C12—C13—C14	−0.45 (12)	C27—C28—C29—C30	0.17 (13)
C12—C13—C14—C15	0.06 (13)	C28—C29—C30—C31	−0.33 (14)
C12—C13—C14—Cl3	−179.04 (6)	C28—C29—C30—Cl6	178.02 (7)
C13—C14—C15—C16	0.28 (13)	C29—C30—C31—C32	0.58 (14)
Cl3—C14—C15—C16	179.39 (7)	Cl6—C30—C31—C32	−177.77 (7)
C14—C15—C16—C11	−0.22 (13)	C30—C31—C32—C27	−0.68 (14)
C12—C11—C16—C15	−0.17 (13)	C28—C27—C32—C31	0.54 (14)
N2—C11—C16—C15	−179.74 (8)	N4—C27—C32—C31	178.16 (8)

### (E)-1-(4-Chlorophenyl)-2-[2,2-dichloro-1-(2,3-dimethoxyphenyl)ethen-1-yl]diazene (II) Hydrogen-bond geometry (Å, º)

**Table:**

*D*—H···*A*	*D*—H	H···*A*	*D*···*A*	*D*—H···*A*
C9—H9*C*···O2	0.98	2.32	2.9059 (12)	117
C10—H10*C*···O3^i^	0.98	2.65	3.3241 (13)	126
C12—H12···O1^ii^	0.95	2.66	3.2919 (11)	125
C25—H25*B*···O4	0.98	2.33	2.9226 (12)	118
C25—H25*C*···N4	0.98	2.58	3.2210 (13)	124
C31—H31···Cl2^iii^	0.95	2.87	3.7991 (10)	166

Symmetry codes: (i) *x*−1, *y*, *z*; (ii) −*x*+1, −*y*, −*z*+1; (iii) −*x*+1, −*y*+1, −*z*+1.
